# Impact of Physical Exercise on Platelets: Focus on Its Effects in Metabolic Chronic Diseases

**DOI:** 10.3390/antiox12081609

**Published:** 2023-08-14

**Authors:** Cristina Barale, Elena Melchionda, Giulia Tempesta, Alessandro Morotti, Isabella Russo

**Affiliations:** Department of Clinical and Biological Sciences of Turin University, Regione Gonzole, 10, Orbassano, I-10043 Turin, Italy; cristina.barale@unito.it (C.B.); elena.melchionda@unito.it (E.M.); giulia.tempesta@edu.unito.it (G.T.); alessandro.morotti@unito.it (A.M.)

**Keywords:** platelets, exercise, obesity, dyslipidemia, diabetes

## Abstract

Chronic disorders are strongly linked to cardiovascular (CV) diseases, and it is unanimously accepted that regular exercise training is a key tool to improving CV risk factors, including diabetes, dyslipidemia, and obesity. Increased oxidative stress due to an imbalance between reactive oxygen species production and their scavenging by endogenous antioxidant capacity is the common ground among these metabolic disorders, and each of them affects platelet function. However, the correction of hyperglycemia in diabetes and lipid profile in dyslipidemia as well as the lowering of body weight in obesity all correlate with amelioration of platelet function. Habitual physical exercise triggers important mechanisms related to the exercise benefits for health improvement and protects against CV events. Platelets play an important role in many physiological and pathophysiological processes, including the development of arterial thrombosis, and physical (in)activity has been shown to interfere with platelet function. Although data reported by studies carried out on this topic show discrepancies, the current knowledge on platelet function affected by exercise mainly depends on the type of applied exercise intensity and whether acute or habitual, strenuous or moderate, thus suggesting that physical activity and exercise intensity may interfere with platelet function differently. Thus, this review is designed to cover the aspects of the relationship between physical exercise and vascular benefits, with an emphasis on the modulation of platelet function, especially in some metabolic diseases.

## 1. Introduction 

Cardiovascular (CV) diseases remain the leading cause of death globally. Physical exercise has been recognized as an important tool to prevent and treat disorders classically associated with increased risk of CV events [1,2]. Actually, regular physical training reduces CV death and rehospitalization in patients affected by coronary artery diseases (CAD) [3,4]. The mechanisms explaining the positive effects of regular physical training are only partially clarified. However, a large body of evidence unanimously indicates regular exercise training as a key tool to improve CV risk factors, including diabetes, dyslipidemia, and obesity [3,5,6]. Notably, each of these metabolic disorders affects platelet function [7], and the correction of hyperglycemia in subjects with diabetes [8] and lipid profile in subjects with dyslipidemia [9,10], as well as lowering the body weight in subjects with obesity [11] all correlate with amelioration of platelet function. Increased oxidative stress due to an imbalance between reactive oxygen species (ROS) production and their scavenging by endogenous antioxidant capacity is the common ground among these metabolic disorders [12]. In particular, when subcellular concentrations of ROS increase beyond homeostatic levels in endothelial cells, the vascular effects of nitric oxide (NO) are compromised, resulting in endothelial dysfunction [13]. Thus, the improvement of platelet function in these dysmetabolic conditions would require a shift in the ROS/NO balance to favor NO within the vasculature.

Metabolic dysfunctions are often characterized by the co-existence of endothelial dysfunction, pro-inflammatory cytokine release, and hypercoagulable state, due at least in part to impaired hemostasis, with a strong interplay with each other.

**Figure 1:**
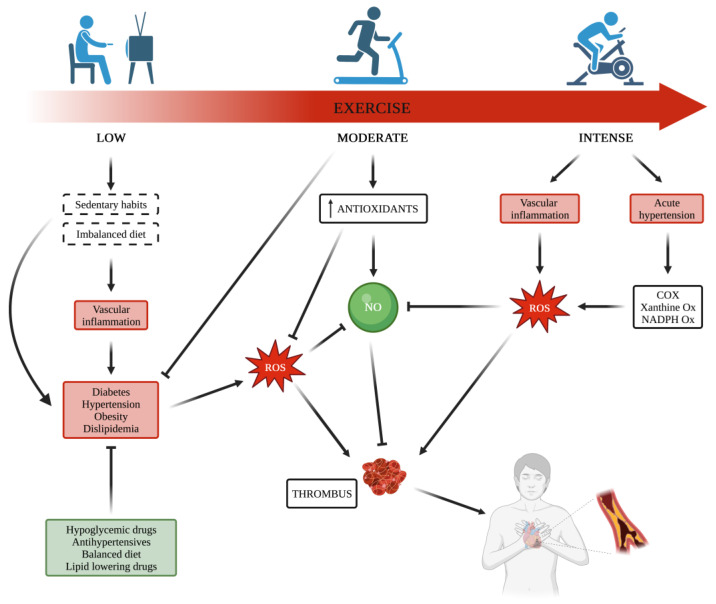
Effects of different types of physical activity on the increased risk of thrombus formation and cardiovascular (CV) events. Sedentary habits induce vascular inflammation that can lead to metabolic diseases. In the same way, prolonged intense training can promote an inflammatory milieu in blood vessels. Reactive oxygen species (ROS) production increases during both types of exercise, reducing nitric oxide (NO) availability and leading to platelet activation, which can contribute to CV events in the long term. Moderate and constant physical activity over time increases the bioavailability of NO, a potent platelet inhibitor, lowering the CV risk. Abbreviations: cyclooxygenase (COX); nicotinamide adenine dinucleotide phosphate (NADPH).

Platelets exert a crucial role in hemostasis and are well-recognized as key players in the development of arterial thrombosis. This role is also closely dependent on platelet ability to release several growth factors and inflammatory molecules involved in the development of CV diseases, which, in turn, are often associated with platelet hyperreactivity. Also, physical (in)activity has been shown to interfere with platelet function. Although data reported by studies carried out on this topic show discrepancies, the current knowledge on platelet function affected by exercise mainly depends on the type of applied exercise intensity and whether acute or habitual, strenuous, or moderate, thus suggesting that physical activity and exercise intensity may interfere with platelet function differently. This is in line with the well-known relationships between physical activity and risk for CV events, where strenuous exercise can increase the risk for myocardial infarction, whereas regular and moderate physical activity significantly decreases the risk of fatal CV events [14,15]. This is an important aspect because the effects of platelet activation are increasingly recognized to be crucial not only for the final step of CV outcomes but also for their involvement in the development and progression of CV dysfunction. Therefore, it is not surprising that there is a mechanistic correlation between the effects of physical activity on platelet function and CV disease-related mortality. Taking into account the important role of exercise in redox status and oxidative stress in the pathways involved in platelet activation, it is reasonable to suppose an association between the protection against CV disease from physical exercise and the effects of exercise on platelet function (Figure 1).

## 2. Platelets in Primary Hemostasis 

**Figure 2:**
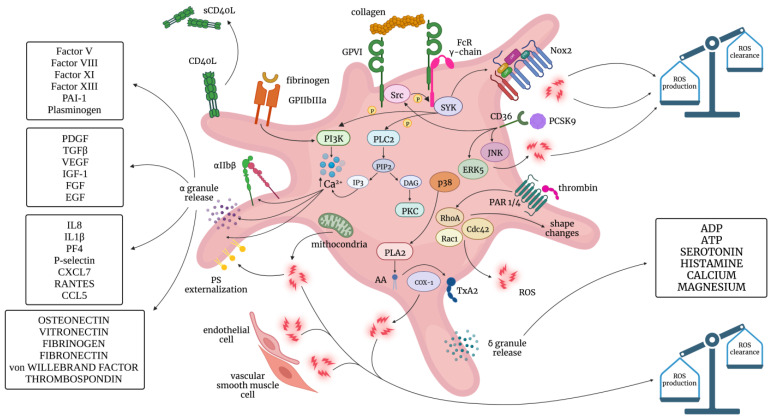
Pathways implicated in platelet activation; molecules involved in platelet granule release and ROS production. Fibrinogen binds to GPIIbIIIa, leading to an increase in cytosolic Ca^2+^ via PI3K; this leads to activation of integrin αIIbβ, granule release and PS externalization. Collagen binds to GPVI, enhancing the downstream FcR γ-chain ITAM domain-SYK pathway. This leads to Ca^2+^ mobilization and increased ROS production through Nox2 activation. The p38-PLA2 pathway leads to TxA2 formation via COX-1; the increase in COX-1 activity leads to ROS formation. Thrombin interacts with PAR1 and PAR4 on the platelet surface. PARs activate the Rho family GTPases RhoA, Rac1, and Cdc42, which are involved in platelet shape changes during platelet activation. This pathway also contributes to ROS generation. PCSK9 binds to the scavenger receptor CD36 on the platelet surface, enhancing ROS production through the GPVI-ITAM-SYK, the ERK5, and the JNK pathways. Other sources of ROS generation are mitochondria and endothelial and vascular smooth muscle cells. The imbalance between ROS production and ROS clearance causes oxidative stress [16].

Platelets are the smallest blood cells, with a lifespan of 7–10 days, and contain different types of secretory granules. Activation of platelets, finally leading to platelet degranulation and aggregation, is a central step for primary hemostasis and coagulation processes and is triggered by a few agonists, including adenosine diphosphate (ADP), thromboxane (TX) A_2_, thrombin, epinephrine, and collagen, as well as by shear and oxidative stress (Figure 2). Indeed, in the process of platelet activation, some of these effectors become part of positive feedback loops in an auto- and paracrine manner.

Platelet adhesion to the injured vessel wall is followed by events involved in the development of both hemostatic plug and thrombus formation. The rheological properties of the blood flow, biochemical components of blood, and vessel blood deeply influence the reactivity of platelets by promoting the release of soluble content of their storage granules as well as by promoting the expression of proteins on the platelet surface as a result of a fusion of membrane granules with platelet membranes. Consequently, activated platelets show features and express proteins that are less detectable in resting platelets, and these can be used as markers of in vivo platelet degranulation/activation. Some of these markers include the platelet index mean platelet volume (MPV) [17], plasma levels of soluble P-selectin (sP-sel), soluble CD40-Ligand (sCD40L), *β*-thromboglobulin (*β*-TG, CXCL7), and platelet factor 4 (PF-4, CXCL4), TXB_2_, Prostaglandin F2 alpha (PGF2α), and the release of platelet-derived microparticles (PMP). Platelet activation correlates with increased plasma expression of P-sel, which is stored in the alpha-granules of platelets, representing the main source of this cellular adhesion molecule with procoagulant activity. If activated, platelets degranulate initially, resulting in the expression of membrane-bound P-sel (CD62P); later, the protein is cleaved off to form a soluble fraction of P-sel. P-sel plays a pivotal role in the development of vascular complications of atherothrombosis [18] and also in its interplay with leukocyte integrins [19]. The measurement of soluble trimeric fragments of CD40L, stored in the cytoplasm in unstimulated platelets and released in a cleaved form within seconds after platelet activation [20], is considered a reliable marker of cardiovascular risk linking thrombosis and inflammation [21]. Platelets, activated by agonists or under high shear stress [22] or increased oxidative stress [23], release PMP, and increased circulating PMP levels correlate with most CV risk factors and indicate a poor clinical outcome. 

If the endothelium is intact and functional, the release of NO and prostacyclin (PGI_2_), the two major physiological antiplatelet agents, prevents thrombus formation inside the blood vessel [24]. When this key protective pathway is overcome, platelet activity becomes uncontrolled. Indeed, circulating platelets are in a quiescent state for NO and PGI_2_ properties to activate cyclic guanosine monophosphate (cGMP)/protein kinase G (PKG) and cyclic adenosine monophosphate (cAMP)/protein kinase A (PKA) pathways, respectively. In conditions of reduced activation of these pathways, platelet sensitivity to agonists increases, contributing to platelet activation [25,26]. 

In clinical settings characterized by increased oxidative stress as a result of an imbalance between ROS synthesis and their neutralization by endogenous antioxidants [27], increased availability of ROS becomes a crucial second messenger, able to deeply influence intracellular signaling also in platelets. Indeed, a large body of evidence shows that both increased ROS generation and their scavenging are involved in the thrombotic process. Metabolic disorders, including dyslipidemia [28,29], metabolic syndrome, and diabetes [7], are all characterized by redox imbalance and the presence of a prothrombotic phenotype.

## 3. Exercise and Cardiovascular Health: Focus on Platelets

The positive correlation between physical activity and CV has been documented by several evidence-based studies. The definition of physical activity often differs, and sometimes this renders the comparison of the results from different studies impossible. Nevertheless, frequency, intensity, and duration of exercise are the three more representative areas of interest in which observational studies have evaluated the complex relationships between exercise and oxidative stress. Intensity refers to the extent of exercise, mainly indicated as a percentage of target heart rate or lung volume. Duration means how long an activity is undertaken, and frequency refers to how often a physical activity is performed. There is agreement on the inverse relationship that exists between physical activity and the occurrence of CV diseases (CVDs). Individuals who perform routine physical activity show a significantly lower risk of disability and a life expectancy about seven years longer than their sedentary counterparts [30,31], and physically active individuals, in comparison with their sedentary counterparts, may have approximately half the incidence of CAD [32], as shown in large multicenter trials carried out on subjects undergone lipid-lowering or antihypertensive therapies [33,34]. A strong, intensity-dependent and inverse relationship between habitual exercise and CAD has also been found in a large survey-based study [35].

Particularly in individuals affected by metabolic diseases such as hypercholesterolemia [36,37], obesity [38,39,40], hypertension [41,42], and diabetes [43,44], exercise training has been demonstrated to be an effective therapeutic intervention able to improve vascular function with a concurrent reduction in mortality for all causes. 

Although the exact mechanisms by which a specific type of exercise acts on the CV system are not completely known, there is agreement on the vascular benefits of exercise on endothelium function originating in the action of an exercise-induced increase in shear stress [45]. Indeed, it has been shown that rhythmic lower limb exercise (cycling and walking) increases artery blood flow and shear rate [as measured by maximal oxygen consumption (VO_2_ max)] [46,47] by mechanisms involving both upregulated expression and activation of endothelial NO synthase [45]. By contrast, maladaptive processes as a consequence of inactivity originating from a sedentary lifestyle are linked to chronically low shear rates determining activation of pathways leading to ROS generation [48,49] and impairment of antioxidant capacity [50] (Figure 1). 

However, a “tipping point” may exist, and a U-shaped curve has been suggested regarding exercise, where the maladaptation to a pronounced sedentary lifestyle or extreme physical activity can be unhealthy with detrimental consequences for NO/ROS balance, favoring ROS increase and impaired NO action at vascular level [51,52,53,54,55]. Indeed, it has been shown that, especially in the high-intensity exercise group, some markers of oxidative stress are increased, thus suggesting that there is a threshold of exercise intensity beyond which ROS generation overcomes the cellular antioxidant capability, favoring a pro-oxidative state [56,57].

Substantial evidence supports the role of exercise intensity as a critical determinant of platelet activation. However, in proposing the concept that exercise can influence platelet function and CV system, we must consider the presence of interindividual differences in terms of response to metabolic stress during physical exercise, for example catecholamine levels, as well as the way to define exercise intensity. In conditions of increased shear stress, platelets are activated but also inhibited by the enhanced endothelial production of NO, which counteracts platelet activation and induces vasodilation for NO effects on vascular smooth muscle cells. While short-term exercise decreases ROS generation and increases antioxidant defense and NO production/bioavailability, regular long-term exercise training causes structural changes to the vessel, leading to an increase in lumen diameter, which in turn decreases shear rates [58,59]. These observations are well-documented and used to recommend regular exercise as a powerful therapy for preventing cardiovascular diseases. Indeed, while the role of platelets in the final step of CV events with thrombus formation is well known, growing evidence suggests the importance of chronic platelet activation in the development and progression of CVD. Therefore, it is not surprising that both acute and chronic exercise, as well as physical inactivity, influence the development of CVD, with similarities in their effects on platelet function [60,61,62]. Finally, extreme physical exercise in healthy subjects may also result in detrimental effects on the cardiovascular system [63], with fewer known effects on platelet function. About 6 to 17% of sudden deaths are caused by vigorous physical exertion [64], and most of these cases are due to the occlusion of the coronary arteries by platelet-rich thrombi [65]. However, the risk is much lower in subjects performing physical activity with regular exertion [66].

The multifaceted beneficial effects of regular exercise on the vascular system and CV outcomes certainly include the improvement of platelet function, which in part derives from a reduction in ROS production and an increase in NO bioavailability (Figure 1). Evidence suggests that exercise training increases NO metabolites and decreases oxidized low-density lipoprotein (oxLDL)-induced platelet adhesiveness on the fibrinogen-coated surface and ADP-induced platelet aggregation [67]. The stimulation of high-density lipoprotein (HDL)-induced prostacyclin production by the endothelium is another mechanism by which exercise, performed regularly, can suppress platelet reactivity [68,69]. In addition, it is useful to remember that platelet activation is closely associated with the release of growth factors, cytokines, and inflammatory mediators, which play essential roles in CVD development. Thus, an improvement of platelet function also means a reduction in proinflammatory and proatherogenic mediators from platelets themselves. Keeping in mind that all CV risk factors favor platelet hyperactivation, it is not surprising that physical (in)activity also influences platelet function.

## 4. Exercise Effects on Platelet Responsiveness 

Regular habitual exercise reduces CVD incidence by improving hemostatic profile both at rest and during exertion. On the contrary, strenuous exercise without training may also lead to sudden cardiac death and venous thromboembolism in subjects with or without underlying vascular disease [15,70,71,72]. Accordingly, with these effects on the CV system, the responses of platelets to different types of exercise change both in healthy and patient populations depending on many variables, including intensity and duration of exercise, and importantly, the physical fitness status of the subject. Moderate-intensity exercise exerts inhibitory effects on platelets, while strenuous exercise stimulates platelet aggregation and activation [73]. While 20-min cycling at 70–80% of the maximal heart rate causes a significant increase in platelet aggregation to agonists, a 12-week regular exercise training blunts platelet hyperreactivity in response to acute exercise in healthy subjects [74]. Accordingly, increased adhesiveness, aggregation, and intraplatelet calcium were observed following an incremental exercise test until exhaustion, whereas these effects were reduced or absent after regular training [74,75,76].

Despite discrepancies among studies, possibly because of methodological differences, it is widely accepted that exercise may increase the rate of oxygen-free radical generation [77,78], which also contributes to skeletal muscle damage and inflammation. Among the biochemical mechanisms involved in the increased production of oxygen free radicals during exercise, two of these generating sources are semiquinone in the mitochondria and xanthine oxidase in the capillary endothelial cells [78]. When the intensity of exercise is high, the oxygen flow through the skeletal muscle cells is greatly increased, and the utilization rate of ATP exceeds the rate of its generation. Usually, the physiological production of free radicals is removed or scavenged by the activity of endogenous antioxidants. When exercise-induced ROS production exceeds the antioxidant capacity, the accumulation of secondary products of lipid peroxidation may occur, thus altering cellular homeostasis [79,80,81,82,83]. For instance, changes in oxidative status cause oxidation of the circulating LDL particles [84] that impact platelet function, causing a predisposition to increased platelet reactivity for increased cyclooxygenase-1 (COX-1) activity and reduced NO production [85]. Considering this, acute exercise without chronic adaptation may paradoxically interfere with platelet responsiveness in a detrimental way through reduced NO bioavailability and ox-LDL generation [75]. Nevertheless, the exact effect of an acute bout of exercise on platelet function is still unclear [86,87].

Even if acute exercise can activate platelets in sedentary healthy subjects, it seems that only vigorous exercise enhances the thrombotic tendency [88,89]. Elevated shear stress and significant release of platelet agonists in plasma during strenuous exercise have been shown to cause platelet aggregation [90,91,92,93,94]. Furthermore, platelets can be influenced by the effects of physical activity on different cell/tissue types and mechanisms. For instance, acute exercise increases the release of catecholamines as well as shear and oxidative stress, all leading to platelet activation. A mechanism leading to a hypercoagulable response to acute exercise is based on catecholamine effects in the mediation of platelet activation [95]. Via alpha2-adrenergic receptors, epinephrine promotes platelet adhesion, aggregation, and binding to fibrinogen [96,97]. While physiological levels of epinephrine may not be enough to trigger the pathways involved in these aspects of platelet response, epinephrine combined with ADP increases after vigorous exercise but not during moderate-intensity exercise [98] and activates platelets in vivo [99], in line with findings indicating platelet hyperreactivity during high-intensity but not moderate-intensity exercise [88,100,101]. It is also important to consider the increase in artery blood flow and shear rate during exercise [47].

Acute vigorous exercise determines a transient increase in platelet count because of hemoconcentration and platelet release by liver, lungs, and spleen [102,103,104]. In addition, platelets released by the spleen are also larger and more active if compared to platelets in the systemic circulation [102,105]. Collectively, the effects of acute exercise on platelet aggregation have been evaluated in many studies with different approaches, and most of them show the tendency to increased platelet aggregability to different agonists after exercise [106,107,108,109,110,111]. Increased platelet aggregation has been found by using optical method in platelet-rich plasma samples [106,107,108,109,110,111] and impedance in whole blood samples in response to agonists [84,112,113,114,115,116,117], and platelet aggregation after shear stress has been found by a rotational viscometer [118,119,120]. However, other studies found no effects [88,121,122,123] or even inhibition of platelet aggregation [124,125,126]. When platelet aggregation was evaluated in whole blood under the combined action of high shear stress and agonists by using the Platelet Function Analyzer (PFA-100), it was reported that after strenuous exercise, closure time values were shorter, thus indicating increased platelet adhesion and aggregation [88,108,127]. Similar results were also observed for platelets in whole blood samples under flow condition using hemostatometry after high- (but not low-) intensity exercise [14].

After acute exercise, a number of studies reported increased plasma levels of *β*-TG [100,110] and PF-4 [100,101], thus representing strong evidence of exercise-induced platelet activation, although this finding is still controversial [88,124,128].

When the role of NO on platelet response to exercise was investigated in physically inactive individuals after moderate and vigorous exercise protocols [68,69], it was supposed that the increased platelet aggregability to agonists found after strenuous exercise, but not after that of moderate intensity, depended on attenuation of platelet sensitivity to NO, possibly due to its lower availability [129]. Indeed, acute high-intensity exertion, but not moderate exertion, induces increased oxidative stress, resulting in enhanced lipid peroxidation products and reduced total antioxidant capacity.

Besides exercise intensity, an important determinant that influences platelet responsiveness to acute exercise is cardiorespiratory fitness; in other words, the adaptation to long-term exercise training. Indeed, regular training improves endothelial [130] and platelet function [73]. If compared with sedentary controls, physically active subjects show higher levels of total antioxidant capacity and reduced platelet reactivity to ADP and collagen. A decreased release of catecholamine as a result of regular physical activity would contribute to attenuating platelet sensitivity to the aggregating effects of epinephrine [131,132].

## 5. Platelets in Exercise Adaptation and Recovery

The efficiency of hemostatic properties and the reduction in thrombotic events following regular exercise and training are largely due to the improvement of endothelial function and vascular tone [133]. Regular training improves both endothelium-dependent and endothelium-independent dilation for more bioavailability of NO, which in turn contributes to contrast platelet aggregation and adhesion [134,135]. 

Indeed, platelet adhesion and aggregation to ADP increase after acute exercise but in trained individuals, this tendency was depressed both at rest and after short-term strenuous exercise [76]. However, the positive effects of training on platelets were reversed back to the pretraining condition after deconditioning [76]. Higher levels of antioxidants and a lower release of adrenaline as well as a reduced platelet membrane expression of alpha2-adrenoreceptors following training are involved in these adaptations that reduce platelet response to common agonists [132]. In light of these, we can suppose that constant platelet inhibition via regular exercise and training may significantly contribute to reducing the risk of thrombotic events at rest and during physical effort [136]. It has also been found that cycling exercise training effectively enhances platelet mitochondrial bioenergetic capacities, including oxidative phosphorylation and electron transport systems, in patients with peripheral arterial disease, with positive effects on their health-related quality of life [137]. Actually, it is well known that lifestyle habits may significantly reduce vascular thrombotic risk. While primary cardiac arrest may transiently increase during vigorous exercise, regular and moderate-intensity exercise reduces the risk of cardiovascular diseases. The mechanisms underlying these different effects on thrombotic processes involve platelets, fibrinolytic, and coagulation activity. Specifically, acute but light exercise (under 49% VO_2_ max) increases fibrinolytic activity but does not interfere with platelet reactivity and coagulation, while if moderate, acute exercise (50–74% VO_2_ max) inhibits platelet reactivity and enhances fibrinolysis without modifying the coagulation system; whereas if vigorous, acute exercise (more than 75% VO_2_ max) activates both platelets and the coagulation system, besides promoting fibrinolytic activity [87,133]. Thus, on the one side, the adaptation to moderate-intensity exercise attenuates platelet reactivity and increases fibrinolysis at rest; on the other side, during strenuous exercise, it limits enhanced platelet reactivity and sustains hyper-fibrinolytic activity. However, the benefits of exercise training on thrombotic patterns are lost after a period of deconditioning. These findings, once again, point out the absolute power of training and appropriate exercise programs in preventing thrombotic events and further cardiovascular disease progression. If endothelial function and its anti-thrombotic properties are impaired as a result of disease or age, it is not surprising that the multi-systemic preventive and therapeutic actions of acute and chronic systemic adaptations of different types of exercise may be lower in comparison with healthy and younger individuals. In any case, for all individuals, with or without existing chronic diseases, it is advisable to initiate exercises at a low-to-moderate intensity and gradually progress to more vigorous ones. 

Interestingly, some studies have also demonstrated the influence of exercise on platelet-rich plasma (PRP) composition and its use in the practice of regenerative medicine [138] and tissue regeneration [139,140,141]. Indeed, different PRP compositions and application protocols result in different implications and therapeutic effects. PRP is a biological concentrate extracted from autologous blood containing a wide variety of substances released by platelets, including platelet-derived growth factor (PDGF), vascular endothelial growth factor (VEGF), transforming growth factor beta-1 (TGF-B1), fibroblast growth factor (FGF), and insulin-like growth factor-1 (IGF-1), which can promote proliferation and differentiation of tissue cells, thus accelerating the process of wound healing [138] and tissue regeneration [139,140,141]. Actually, growth factors act in many stages of the wound-healing process, regulating the growth and differentiation of cells involved in the regeneration of tissues and blood vessels and contributing to angiogenesis, inflammation, and coagulation. Generally, physical therapy and rehabilitation programs represent effective strategies in getting athletes back to sport, and the return-to-play time depends on the injury severity. However, basic research and preclinical data indicate the use of PRP for sports-related injuries, given that injections of PRP in some situations have been shown to be effective in aiding the healing process, favoring exercise recovery and repair of muscle microinjuries [142]. Although research regarding its clinical efficacy is still in the early stages and it is not known whether PRP injections have any effect on the risk of new hamstring injuries, some athletes already use PRP in the treatment of sports-related injuries [143]. Regarding the optimal formulation of PRP, both a short-time bout of high-intensity interval exercise and longer duration exercise are able to significantly increase platelet count and the number of growth factors [144]. Nevertheless, the specific changes that occur in PRP extracted after resistance training are not extensively characterized and warrant further interrogation.

## 6. Metabolic Diseases and Exercise Effects on Platelets

Metabolic disorders, including obesity, dyslipidemia, and diabetes are well established risk factors for CVDs [145], being associated with underlying abnormalities that trigger some common biochemical events influencing platelet response, such as enhanced ROS formation, decreased availability and/or synthesis of NO, lipid peroxidation with the consequent increased production of TXA_2_ and free radicals, and non-enzymatic–catalyzed generation of bioactive isoprostanes, which activate TX receptor [11,146,147].

In each of these metabolic disorders, it is reasonable to suppose that physical activity, with or without weight reduction, reduces cardiometabolic disorder risk, partially by improving insulin sensitivity and lowering blood pressure [148,149]. Indeed, among the biological mechanisms by which exercise confers its benefits, the increased sensitivity to both the metabolic [150,151] and vascular [152] actions of insulin may play an important role. Platelet membrane expresses insulin receptors with a density similar to that of cell types of the targets of the metabolic actions of the hormone [153]. Platelet insulin receptors activate the intracellular pathway classically linked to insulin signaling, even in the absence of a response of glucose uptake [154]. Obese insulin-resistant subjects with or without diabetes show impaired platelet responsiveness to the inhibitory effects of insulin [25,155], thus suggesting that each strategy useful in reducing insulin-resistance can also improve the vascular effects of insulin including its antiplatelet effects.

***Platelet Alterations in Obesity.*** One of the first epidemiological studies showing a strong correlation between obesity and CV events was the Framingham Heart Study [156,157]. From then, many other studies have confirmed the role of waist-to-hip ratio (WHR), an index of central obesity, as the strongest anthropometric predictor of myocardial infarction [158] and stroke [159,160].

The release of cytokines and free fatty acids from abdominal adiposity has a causal, unfavorable effect on lipid profile and other cardiometabolic risk factors involved in the pathogenesis of both atherothrombosis and insulin resistance [161,162]. Chronic low-grade inflammation and systemic oxidative stress have both been associated with obesity causing endothelial dysfunction with the consequent loss of its antithrombotic properties and arterial damage, thus justifying the assumption that obesity is a pro-thrombotic condition due to vascular disease, increased platelet activation, and hypercoagulability [163,164,165,166,167]. Adipose tissue is an important source of ROS and the increased level of systemic oxidative stress contributes to the development of obesity-associated insulin resistance and type 2 diabetes mellitus (T2DM) and other disorders, such as hypertension, atherosclerosis, and cancer [168,169]. Excess intake of nutrients, a sedentary lifestyle, and the consequent weight gain promote ROS production and mitochondrial dysfunction [170], a risk factor for T2DM, atherosclerosis, and hypertension [171]. Among the in vivo parameters of platelet activation, MPV, a marker closely related to platelet hyperactivation, has been found to be increased in obesity [17,172,173], and a positive correlation also exists between MPV and body mass index (BMI) after weight loss [172,174,175]. A platelet activation marker associated with obesity is also sP-sel, which is able to predict atherosclerosis independently of BMI and other CVD risk factors [176]. The increased circulating levels of sP-sel found in overweight and obese subjects [11,177] are reduced after weight loss [11]. Obese subjects show increased levels of 11-dehydro-TXB_2_ and PGF_2α_, thus underlining the link between platelet activation and oxidative stress [178]. Indeed, the chronic ‘metabolic inflammation,’ which is considered the hallmark of obesity and causes insulin resistance and T2DM [179], significantly contributes to increases in the systemic levels of ROS, which affect platelet reactivity by different mechanisms, including decreased NO bioavailability, increased expression of membrane glycoproteins, impairment of calcium mobilization, and isoprostane generation [178]. In obesity, in comparison with non-obese subjects, elevated PMP levels positively correlate with BMI and waist circumference [180], although this finding was not confirmed in other studies, where PMP did not appear to differ in number [181] but were greatly heterogeneous in size and distribution, with different levels of proteins involved in thrombosis and tumorigenesis [181].

In our previous studies, we provided evidence of persistent platelet hyporesponsiveness to NO and PGI_2_ pathways in obesity and T2DM [26,182,183]. We demonstrated the presence of multi-step defects at the level of NO/cGMP/PKG and PGI_2_/cAMP/PKA pathways. Specifically, platelets from obese subjects show an impairment in the respective abilities of NO and PGI_2_ to increase cGMP and cAMP synthesis, and resistance of cGMP and cAMP themselves in activating their specific kinases PKG and PKA [26,182]. As these are cyclic nucleotides effective in reducing intracellular Ca^2+^ [184], our data explained one of the mechanisms implicated in Ca^2+^ flux alterations found in insulin-resistance states [185] and the defective action of cyclic nucleotides on platelet function. In addition, hyperglycemia does not emphasize this multistep resistance [186], and the presence of diabetes without obesity is not associated with platelet abnormalities observed in obese subjects [186]. These findings support the hypothesis that the abnormalities leading to platelet hyperreactivity are mainly related to the underlying metabolic disorders dependent on visceral adipose tissue activity rather than on platelet exposure to hyperglycemia effects. Besides changing subcutaneous and visceral adipose tissue distribution, insulin sensitivity, and beta-cell performance, a dietary program aiming at achieving weight loss of at least 7–10% of initial body weight leads to a significant reduction in systemic inflammation, oxidative stress, lipid peroxidation, and platelet reactivity [11].

***Exercise Effects on Platelets in Obesity.*** A recent systematic review and meta-analysis including 25 randomized controlled trials (1686 participants) shows that regular aerobic exercise significantly decreases visceral adipose tissue with more pronounced benefits for higher intensity exercise [187]. It has also been ascertained that independently of age, body mass index, and exercise training characteristics, aerobic training in adults with overweightness or obesity and with cardiometabolic disorders is effective in reducing postprandial glucose and insulin levels [188]. As far as platelet parameters are concerned, a randomized clinical trial performed in overweight men showed that moderate-intensity training for 12 weeks consisting in walking/slow jogging exercise at 45–55% of VO_2_ max (5x/week for 45–60 min) led to a reduction in platelet aggregation associated with a reduction in serum TXB_2_ levels [135]. To determine the role of exercise in platelet reactivity in obese patients with coronary artery disease, a 4-month program of training exercise and behavioral weight loss was performed by Keating et al. [189]. These authors found a significant decrease in P-sel expression not independently associated with measures of body composition or fitness. After controlling for exercise group and gender, the change in platelet reactivity was more pronounced in females and associated with changes in high-sensitivity C-reactive protein and a reduction in insulin-resistance. A study carried out on blood samples taken from obese women before and immediately after exercise demonstrated that vigorous aerobic exercise, consisting of a 30-min walking exercise test at an intensity of 70% of individual peak oxygen uptake, was able to significantly prolong the clot formation time as measured by thromboelastometry and reduce the fibrin buildup after exercise. Thrombography revealed a significant exercise-induced decrease in endogenous thrombin potential [121]. On the basis of these results, the authors postulated that vigorous aerobic exercise might be a suitable strategy to protect obese women from thrombotic events. Indeed, this assertion has been confuted in favor of regular exposure to high-intensity exercise in order to desensitize against exercise-induced platelet aggregation, attenuate coagulatory parameters, and up-regulate fibrinolytic potential [87]. In another study, obese subjects underwent moderate-intensity exercise on a treadmill (at 60% of their VO_2_ max), and the results showed changes in size distribution and cell origin of extracellular vesicles (EVs) [190]. Total EVs, exosomes, and CD61+ EVs were significantly associated with HOMA-IR, and flow cytometry assays revealed that acute exercise provided a significant improvement of hemostasis parameters, including reduced platelet aggregability [191] (Table 1).

***Platelet Alterations in Dyslipidemia.*** Dyslipidemia promotes the atherosclerosis process because of the chronic accumulation of lipid-rich plaque in arteries [192], and its relationship with the increase in CV risk depends on its long-term effects on atherogenesis as well as on its influence on thrombogenesis [28]. Lipid profile alterations are associated with increased oxidative stress, and the generation of oxidized lipids, such as ox-LDL, leading to platelet hyperreactivity [16,193]. In turn, activated platelets can generate ox-LDL, thus contributing to propagating platelet activation, and inducing thrombus formation through oxidative stress-mediated mechanisms. In particular, the ROS-producing enzyme nicotinamide adenine dinucleotide phosphate (NADPH)-oxidase (NOX)-2 (NOX2)-dependent increase in oxidative stress is involved in platelet ability to propagate the oxidation of lipoproteins [194,195]. Oxidized lipoproteins are deeply involved in many biochemical events leading to atherosclerosis processes as well as platelet hyperactivation [196,197].

Platelet hyperreactivity, strongly and independently associated with thrombotic events [198,199,200,201,202], is characterized by redox imbalance [9,10,203]. Mechanistically, ROS and oxidation reactions are, per se, the cause of vascular dysfunction [168,204,205], but dyslipidemia also increases the risk of CVDs due to the effects of plasma oxidized lipids on platelet function [206] via the interaction of ox-LDL with scavenger receptors, such as CD36, and signaling pathways, including the Src family kinases (SFK), mitogen-activated protein kinases (MAPK), and NADPH-oxidase. 

In their native form, LDL particles do not increase platelet aggregation, whereas their oxidative modifications make these lipoproteins able to act as aggregating agents in the absence of physiological agonists [207]. In hypercholesterolemia, the loss and/or impaired effects of NO on platelets are important determinants for platelet hyperactivation. The increased oxidative stress causes a decrease in NO bioavailability [208] and a decreased sensitivity to NO-related pathways [11,209,210,211]. Additionally, a lower NO-mediated inhibitory effect of the incretin hormone glucagon-like peptide 1(GLP-1) [212] has been found as a putative mechanism by which hypercholesterolemia can induce platelet hyperactivation [209]. Patients with hypercholesterolemia show increased levels of TXA_2_, superoxide anion, and platelet activation markers, including sP-Sel, PF-4, sCD-40L, and β-TG [9,10,203]. Importantly, the reductions in oxidative stress-related abnormalities obtained through pharmacological interventions can significantly improve platelet function. Indeed, many platelet alterations significantly improve after treatment with classical lipid-lowering drugs, such as statins [10,81,213,214,215,216], and the more recent and aggressive therapies, such as the anti-proprotein convertase subtilisin/kexin type 9 (PCSK9) antibodies [9,217].

***Exercise, Lipid Metabolism, and Platelets***. Exercise has positive impacts on reducing cholesterol levels and improving the physical fitness of individuals with dyslipidaemia [218]. A significant positive association exists between exercise and HDL cholesterol, while a significant negative association has been reported between exercise and triglyceride levels, total cholesterol, LDL cholesterol, and triglycerides after a 5-year follow-up [219]. It is generally accepted that regular physical exercise, with a linear dose–response relationship, increases HDL cholesterol while regulating and theoretically preventing increases in LDL cholesterol and triglycerides [220]. More intense activity seems required to obtain significant reductions in LDL cholesterol and triglyceride levels, even if a bout of prolonged aerobic exercise has been shown to be effective in lowering blood postprandial hypertriglyceride levels in individuals at high risk of developing CVD [221]. Indeed, exercise modality, cardiovascular exercise type, and timing of exercise can vary in their attenuation of postprandial triglyceride levels depending on exercise energy expenditure prior to meal administration [222]. 

As known, exercise increases oxygen consumption with the consequent increase in oxygen-related free radicals. The increased exercise-mediated oxidative stress can induce lipid peroxidation, membrane damage, and platelet activation due to the effects of both native LDL and LDL modified under oxidative stress on platelets [223,224]. The deleterious effect of strenuous exercise on platelet activity has been confirmed by a study carried out on healthy subjects showing an increase in plasma TX level at peak exercise and its return to pre-exercise levels at 10 min postexercise [84]. Intriguingly, this study also showed that a treadmill exercise test to the point of physical exhaustion induced platelet aggregation, increased TX, *β*-TG, and lipid peroxide levels. However, that acute exercise decreased LDL lipid peroxides, reaching a statistically significant lower plasma concentration at 10 min post-exercise. The authors speculated that during strenuous exercise, LDL lipid peroxides can replace plasma LDL cholesterol (LDL-C), attenuating the role of LDL on platelet activation. To explain this paradoxical result, ex vivo experiments were also performed by adding mildly oxidized LDL to peak exercise blood. The result was a decrease in platelet aggregation, suggesting that LDL lipid peroxides attenuate exercise-induced platelet aggregation. However, the question of why mildly ox-LDL, in conditions of strenuous exercise, attenuated instead of stimulating platelet aggregation remains to be explored.

In another study, sedentary individuals performing exercise training for 8 weeks failed to decrease their circulating ox-LDL levels but reduced plasma total cholesterol and LDL-C levels and positively influenced platelet function, as demonstrated by the reduced ability of ox-LDL added in vitro to increase the agonist-induced aggregation and intraplatelet calcium elevation in blood samples collected at both resting and postexercise [75]. However, detraining reverses the benefits of training on lipid profile and platelet function, and in contrast to regular, strenuous acute exercise, it increases platelet aggregation and calcium elevation promoted by 100 microg/mL of ox-LDL [75]. These findings confirm the positive effects of the adaptation to long-term exercise training.

Besides lipid profile amelioration, the high-fat diet combined with the swimming group is able to improve many hemostasis parameters, including platelet reactivity, as shown by prolonged bleeding time, reduced platelet aggregability and spread of fibrinogen, and decreased activation of pathways implicated in platelet activation [225].

The effects of an 8-week high-intensity aerobic exercise on in vivo lipid peroxidation and platelet activation were investigated in healthy sedentary individuals with low HDL cholesterol levels. Exercise training did not modify total cholesterol or LDL-C concentrations but significantly reduced oxidative stress (8-iso-PGF_2α_) and platelet activation (11-dehydro-TXB_2_) urinary markers [226] (Table 1).

***Platelet Alterations in Diabetes.*** Several studies provide evidence of the enhanced activation of platelets in T2DM [227,228,229,230,231]. Increased values of MPV, an indicator of platelets larger in size and metabolically more active, and platelet distribution width (PDW) are indicative of platelet activation and associated with thrombotic events [232]. The consequent platelet hyperreactivity triggers the release of multiple molecules stored in α-granules, dense granules, and lysosomal granules.

The persistent platelet activation represents an important link between diabetes and atherothrombosis, although evidence from the literature shows platelet activation already in prediabetes [233] or newly diagnosed T2DM patients with central obesity in good metabolic control [234]. Indeed, lipid peroxidation and TX-dependent platelet activation, as mirrored by in vivo urinary excretion of PGF_2α_ and 11-dehydro-TXB₂, correlate with atherothrombosis from the earlier stages of T2DM [234]. Nevertheless, a linear correlation was observed between the urinary excretion of the stable TX metabolite 11-dehydro-TXB2 and either body mass and plasma fasting or postprandial glucose. The exact role of adiposity, adipose tissue inflammation, insulin resistance, and hyperglycemia in persistent platelet hyperreactivity in diabetes is difficult to clarify. Hyperglycemia is not a strong risk factor for CVD [235,236], as confirmed by the evidence that interventions aimed at reducing plasma glucose did not significantly reduce CV risk and mortality [237,238,239]. Consistently, the pharmacological reduction in glycated hemoglobin (HbA1c) only modestly improved CVD risk and mortality [235,239], whereas newer drugs, including GLP-1 agonists and gliflozins, beyond their glucose-lowering effects, have provided effective results in terms of reduction in CV risk [240,241,242], thus indicating the need to modulate risk factors other than hyperglycemia to blunt atherothrombosis [243].

***Exercise Effects on Platelets in Diabetes.*** Besides cardio-pulmonary fitness and weight control, exercise training improves glycemic control and insulin-resistance in T2DM [244] and is strongly recommended for its benefits on the CV system [245]. A total of 106 randomized controlled trials involving 7438 patients were included in a recent meta-analysis aimed at evaluating exercise effects in adults with T2DM. In comparison with no exercise, low to moderate supervised aerobic/resistance exercise is associated with significant improvement of glycemic and lipid profile, body weight, and blood pressure [187]. 

Regular physical exercise in diabetes also shows beneficial effects on platelet function (Table 1). Specifically, aerobic training for 8 weeks determines a remarkable reduction in MPV, PDW, and collagen-induced platelet aggregation [246], attributable at least in part to downregulated glycoprotein (GP)IIb expression. A 12-week moderate-intensity aerobic exercise program was effective in upregulating platelets’ microRNAs (miRNA)-223 and downregulating P2RY12 receptor expression following decreased platelet aggregability in T2DM patients [247], whereas short-term endurance training determined a positive impact on platelet function, glycemic indices, physical fitness, and body composition, but did not change miRNA-223 levels and P2RY12 expression [248]. One year of exercise training was not effective in modifying platelet-derived microvesicles in T2DM patients with CAD, but decreased levels of PMVs carrying TF [CD61^+^/CD142^+^/Annexin V (AV)^+^] and von Willebrand factor (vWF; CD31^+^/CD42b^+^/AV^+^) in those with albuminuria [249]. In another study, acute exercise increased platelet aggregation in diabetic subjects despite treatment with aspirin diabetics, thus showing the limited effects of aspirin in inhibiting exercise-induced platelet aggregation [250]. The impaired action of aspirin could be partially explained by taking into account that endothelial dysfunction caused by inflammation and oxidative stress causes an impaired release of PGI2 and NO following acute exercise, thus limiting the antiplatelet effects of aspirin [251]. Another randomized crossover design evaluated the short-term effects of post-meal walking exercise with and without a low-carbohydrate diet on vascular parameters. The authors found that a 15-min post-meal walk in addition to a diet significantly improved endothelial function, even if its role in platelet reactivity, as measured through PMP release and monocyte platelet aggregate (MPA) count and percentage, was unclear [252]. The effects of postprandial hyperglycemia in impairing endothelial function and increasing oxidative stress are particularly concerning for their role in the excessive CVD risk in diabetes [253,254], and the correction of hyperglycemia and oxidative stress can positively influence endothelial function, at least in an acute setting [255]. A limitation of this study was certainly its short duration, which did not allow the observation of the endothelium-mediated benefits for platelets. In a study carried out by Scheinowitz et al., diabetic patients in antiaggregating therapy with aspirin were enrolled and undertook acute exercise. Platelet samples at rest and immediately post-exercise were stimulated with agonists, and the expression of the pan-platelet marker CD41 and platelet activation marker CD62P was measured [256]. Despite diabetic patients showing systolic blood pressure significantly higher than non-diabetics, no differences were found in platelet parameters. Finally, platelet CD markers of platelet activation did not change in a study comparing the effects of blood-flow restriction under low-intensity resistance exercise (20%) versus high-intensity resistance exercise (80%) in female T2DM patients, even though CD62P, CD61, CD41, and CD42 were reduced following resistance exercise in both trials independently of blood-flow restriction conditions [257] (Table 1).

**Table 1:** Exercise and platelet parameters in metabolic diseases. Abbreviations: adenosine diphosphate (ADP); adenosine triphosphate (ATP); thromboxane B2 (TXB2); platelet (PLT); coronary artery disease (CAD); cardiac rehab (CR); high caloric CR (HCR); extracellular vesicles (EVs); exercise (EX); high carbohydrate and fat diet (HCFD); high-fat (HF); high-fat + exercise (FE); oxidized Low-Density Lipoprotein (ox-LDL); prostacyclin (PGI2); coronary heart disease (CHD); high-density lipoprotein cholesterol (HDL-c); prostaglandin F2α (PGF2α); mean platelet volume (MPV); platelet distribution width (PDW); plateletcrit (PCT); type 2 diabetes mellitus (T2DM); monocyte-platelet aggregates (MPAs); blood-flow restriction (BFR).

Study Design	Population	Number of Individuals	Exercise Protocol	Platelet Parameters	Effects of Exercise	**Reference**
**Obesity**						
Randomized controlled trial	Men	53	Progressive training program for 12 weeks	ADP-induced platelet aggregation	↓ ADP-induced PLT aggregation (Ex group vs. Ref group)	[135]
		Exercise group: 26	Sessions: five times/week, 45 to 60 min per session.	Release of ATP	No significant change	
		Reference group: 27		Serum TXB2	↓ TXB2 (Ex group vs. Ref group)	
**Prospective randomized trial**	CAD	46 Cases CR: 21 standard cardiac rehab (CR) Cases HCR: 25 high caloric CR	All: 4 months of intervention + 1 month of weight stabilization with training CR: 3 sessions/week of 25 to 40 min HCR: 5 to 7 sessions/week of 45 to 60 min	P-Selectin expression GPIIb/IIIa activation	↓ P-Selectin expression (5 months vs. baseline, all subjects) ↓ P-Selectin expression (5 months vs. baseline, HCR) GPIIb-IIIa activation no significant change (5 months vs. baseline, all subjects)	[189]
Controlled clinical intervention study	Women	42	All: 30-min walking exercise test with an intensity of 70% of individual peak VO_2_	Thrombus formation Collagen-induced platelet aggregation Platelet adhesion	↑ clot formation time (post vs. pre) ↓ alpha-angle (post vs. pre) No significant change	[121]
				PLT count	No significant change	
Case-control study		23	All: in two different days two exercise protocols	PLT EVs (CD61+)	↓ PLT EVs (postexercise 24 h vs. basal, all subjects)	[190]
		Cases: 15	Pilot test: incremental exercise on a treadmill until voluntary exhaustion			
		Controls: 8	Submaximal test: 30 min of moderate constant workload			
**Randomized controlled trial**	Rats	24	All: 15 weeks	ADP-induced platelet aggregation	↓ ADP-induced PLT aggregation (HCFD + Ex vs. HCFD)	[191]
		Cases Ex: 6	Group Ex: swimming 3 days/week, 1 h	PLT count	No significant change	
		Cases HCFD: 6	Group HCFD: high-fat diet			
		Cases HCFD + Ex: 6	Group HCFD + Ex: high-fat diet + swimming from the 11th week to the 15th week			
		Controls: 6	Controls: no exercise training			
**Lipid Profile** **Alterations**						
**Randomized controlled trial**	Mice	63	All: 8 weeks	PLT aggregation rate	↓ PLT aggregation rate (FE vs. HF)	[186]
		Controls: 21	Cases HF: high fat diet	PLT spread on fibrinogen	↓ PLT spread on fibrinogen (FE vs. HF)	
		Cases HF: 21	Cases FE: swimming 60 min/day, 5 days/week	PLT pAKT level	↓ PLT pAKT level (FE vs. HF)	
		Cases FE: 21				
**Randomized controlled trial**	Sedentariety	10	1. Strenuous, acute exercise	ADP-induced PLT aggregation	↑ ADP-induced PLT aggregation (strenous ex vs. rest)	[75]
			2. Ergometer cycling:	ADP-induced [Ca^2+^]_i_ elevation	↑ ADP-induced [Ca^2+^]_i_ elevation (strenous ex vs. rest)	
			30 min/day, 5 days/week, 8 weeks	ox-LDL-induced PLT aggregation	↑ ox-LDL-induced PLT aggregation (strenous ex vs. rest)	
			3. 12 Weeks detraining	ox-LDL-induced [Ca^2+^]_i_ elevation	↑ ox-LDL-induced [Ca^2+^]_i_ elevation (strenous ex vs. rest)	
					↓ ADP-induced PLT aggregation (training vs. pre-training)	
					↓ ADP-induced [Ca^2+^]_i_ elevation (training vs. pre-training)	
					↓ ox-LDL-induced PLT aggregation (training vs. pre-training)	
					↓ ox-LDL-induced [Ca^2+^]_i_ elevation (training vs. pre-training)	
					↑ ADP-induced PLT aggregation (detraining vs. training)	
					↑ ADP-induced [Ca^2+^]_i_ elevation (detraining vs. training)	
					↑ ox-LDL-induced PLT aggregation (detraining vs. training)	
					↑ ox-LDL-induced [Ca^2+^]_i_ elevation (detraining vs. training)	
**Randomized controlled trial**	Healthy	30	Treadmill test using Bruce protocol	Plasma TX levels	↑ plasma TX levels (pre vs. post)	[84]
				Plasma PGI_2_ levels	No significant change	
				After ex vivo addition of mildly ox-LDL:		
				TX release	↓ TX release (mildly ox-LDL+ vs. mildly ox-LDL-)	
				Collagen-induced PLT aggregability	↓ collagen-induced PLT aggregability	
					(mildly ox-LDL+ vs. mildly ox-LDL-)	
**Cross-sectional study**	CHD	18	All: 8 weeks	Urinary 8-iso-PGF2a	↓ Urinary 8-iso-PGF2a (post vs. pre)	[219]
	Sedentariety		2 sessions/week on cycle ergometer	Urinary 11-dehydro-TXB2	↓ Urinary 11-dehydro-TXB2 (post vs. pre)	
	Low HDL-c		55 min/session, supervised			
**DIABETES**						
**Randomized controlled trial**		24	All: 12 weeks	ADP-induced PLT aggregation	↓ ADP-induced PLT aggregation (Cases vs. Controls)	[240]
		Cases: 12	Cases: walking and running on the treadmill in Non-consecutive days	miRNA-223 expression	↑ miRNA 223 expression (Cases vs. Controls)	
		Controls: 12	Controls: no exercise training			
**Quasi-experimental controlled trial**	Sedentariety	24	All: 8 weeks	Collagen-induced PLT aggregation	↓ collagen-induced PLT aggregation (post vs. pre, cases)	[239]
			Cases: mean intensity treadmill, 3 times/week	PLT, MPV, PDW, PCT	↓ MPV, ↓ PDW (post vs. pre, cases)	
			Controls: no exercise training	Glycoprotein IIb (GPIIb) receptor expression	Down-regulated (post vs. pre, cases)	
				miR-130a expression	No significant change	
**Randomized controlled trial**	CAD	74	All: 12 months	PMVs	No significant change	[242]
	Albuminuria (n = 25)	Cases: 38	Cases: aerobic and resistance training	PMVs in patients with albuminuria	↓ PMVs carrying TF (CD61+/CD142+) (post vs. pre, cases)	
		Controls: 36	150 min/week		↓ PMVs carrying vWF (CD31+/CD42b) (post vs. pre, cases)	
**Prospective study**	Aspirin treatment	79	All: single treadmill exercise test	PLT aggregation (ASPI test)	Cases: ↑↑ PLT aggregation (post vs. pre)	[243]
	Cases: T2DM	Cases: 43			Controls: ↑ PLT aggregation (post vs. pre)	
	Controls: no T2DM	Controls: 36				
**Randomized crossover study**		11	All: post-meal walks	PMVs	No significant change	[245]
			15 min/day	MPAs	↑ MPAs (post vs. pre, cases)	
			4 days			
**Randomized controlled trial**	Cases: T2DM	16	1 session of nuclear exercise stress test	PLT count	No significant change	[249]
	Controls: no T2DM	Cases: 8	Bruce protocol	ADP-induced PLT activation	No significant change	
		Controls: 8		Collagen-induced PLT activation	No significant change	
				Arachidonic acid-induced PLT activation	No significant change	
				Aspirin responsiveness	No significant change	
**Randomized controlled trial**	Women	20	All: 8 weeks	ADP-induced platelet aggregation	↓ ADP-induced PLT aggregation (Cases vs. Controls) trend *p* = 0.06	[241]
		Cases: 10	Cases: Endurance training, 3 non-consecutive days/week	miRNA-223 expression	↑ miRNA 223 expression (Cases vs. Controls) trend *p* = 0.06	
		Controls: 10	Controls: no exercise training			
**Randomized crossover study**	Women	15	All: 2 resistance exercises	P-Selectin expression	No significant change	[250]
		Cases: training with blood-flow restriction (BFR)		GPIIb/IIIa activation CD42 expression	No significant change No significant change	
		Controls: training w/o BFR		CD61 expression	↓ CD61 expression (Controls post vs. pre)	
				PLT count	↑ PLT count (Cases vs. Controls)	
				Plateletcrit (PCT)	↑ PCT (Controls vs. Cases)	
				PDW	No significant change	
				MPV	No significant change	

## 7. Discussion

Atherothrombotic events leading to an increased risk of CV morbidity and mortality are closely associated with cardiometabolic disorders, including central obesity and impaired lipid/glucose metabolism. Subjects with these disorders show ‘angrier’ platelets because they show several abnormalities, which increase platelet aggregability and activation. The studies reviewed included the effects of both short-term strenuous and long-term exercise on platelet function and activation. The majority of studies, independently of type of metabolic disorder, indicated the beneficial effect of prolonged moderate-intensity training exercise on platelets in terms of aggregation and activation [75,135,189,226,246,247,249]. The effects of acute strenuous exercise on platelet response were contradictory. For instance, vigorous aerobic exercise in obese women improved hemostasis parameters with prolongation of clot formation in one study [121]. In contrast, strenuous acute exercise increased platelet aggregation and activation [75,84] through an LDL (but not oxLDL)-dependent mechanism in non-obese subjects [84]. Curiously, in contrast to other studies [207,217], an inhibitory effect of mildly ox-LDL particles on platelet aggregation was found [84]. 

The role of adaptation to long-term exercise in platelet function and the role of lipid metabolism in regulating platelet response emerged from the elegant study by Wang et al. [75]. On the one hand, they demonstrated that detraining blunts the benefits deriving from regular training in reducing platelet sensitivity to the pro-aggregating effects of ox-LDL; on the other hand, they also found lower platelet aggregation and activation in samples collected at resting and after acute exercise in training in comparison with pretraining state. Indeed, the role of exercise in improving platelet function in altered lipid profile was confirmed in high-fat-fed animal models as well as in subjects with low HDL cholesterol. The decrease in markers of oxidative stress (8-iso-PGF2α), obtained not only by applying dietary or pharmacological intervention but also by physical exercise, remains an important mechanism by which platelet function may significantly improve, as demonstrated by Vazzana et al. [226]. In T2DM, platelet activation following one session of nuclear exercise stress test persisted despite aspirin therapy [256] as well as after 15-min post-meal walking [252]. The impaired endothelial function due to redox imbalance and inflammation associated with diabetes could in part explain the lack of effects of short-time exercise on platelet function. 

This review provides details of study populations, the methods used to evaluate platelet function or activation, and the main findings obtained. The reviewed studies included a control group and were prospectively designed, reducing the risk of bias. However, we have to consider some limitations. The characteristics of patients and controls varied within each clinical setting and only a small number of subjects were included in the studies. 

## 8. Conclusions

Despite some contradictory reports, most studies suggest that exercise can influence different aspects of platelet function, and the different methodological approaches used to investigate platelets may at least in part explain some contradictory conclusions. The intensity of the applied exercise remains a crucial determinant of the effects on platelet function, which is influenced by both acute exercise and regular training. Platelet hyperreactivity is a common feature of obesity, dyslipidemia, and diabetes and, in each of these dysmetabolic states, exercises at a low-to-moderate intensity seem able to reduce platelet activation and response to agonists. However, more investigations are needed in this field. Controlled randomized trials with appropriate sample size and standardized measurements are essential requisites in these studies. Although the specific ‘antiplatelet effects’ of regular exercise training are not clearly defined, the role of a reduction in oxidative stress and its related products in the improvement of platelet function in metabolic diseases has been established, explaining, at least partially, the benefits of routine moderate physical activity for the CV system.

## Data Availability

Not applicable.
